# Identification and validation of myocardial infarction and stroke outcomes at scale in UK Biobank

**DOI:** 10.23889/ijpds.v1i1.358

**Published:** 2017-04-19

**Authors:** Christian Schnier, Spiros Denaxas, Rosalind Eggo, Riyaz Patel, Qiuli Zhang, Rebecca Woodfield, Robin Flaig, Harry Hemingway, Cathie Sudlow

**Affiliations:** 1 UK Biobank / Edinburgh University; 2 Farr Institute of Health Informatics Research, London; 3 London School of Hygiene and Tropical Medicine

## Aims

We aimed (i) to create algorithms for identifying stroke and acute myocardial infarction (MI) cases in a large prospective population-based study (UK Biobank), using linked data from national hospital admissions data and death registers, combined with self-report data from the baseline assessment; and (ii) to assess validity by examining associations with risk factors and mortality.

## Methods

UK Biobank is a prospective study of 503,000 participants, aged 40-69 years when recruited in 2006-2010 from centres across England, Scotland and Wales. Participants provided extensive questionnaire data on lifestyle, environment and medical history (with confirmation of self-reported medical conditions during a brief interview with a trained research nurse), had physical measures, and provided biological samples. Follow-up is principally through linkages to national health-related datasets, integrated from different data providers for each country and including cohort-wide data for ICD-coded hospital admissions and registered deaths, with follow-up to 2011 for this report. We used expert opinion and systematic reviews to identify baseline self-report items and ICD codes maximising positive predictive value for identification of stroke, MI and their subtypes. We classified participants with at least one relevant code as being first affected either before (prevalent cases) or after recruitment (incident cases). We compared cases with non-cases (controls) using logistic regression (prevalent cases) and survival analysis (incident cases) to examine associations with vascular risk factors, defined using data from the baseline assessment. We used survival analysis to compare vascular, non-vascular and all-cause mortality for cases versus controls post recruitment (prevalent cases) and post hospitalization (incident cases).

## Results

We identified 8654 stroke cases and 13,479 MI cases. 90% of both were prevalent at recruitment, of which 29% (stroke) and 54% (MI) were identified through both research nurse-confirmed self-report and hospital admission records. During ≈1 million person years of follow-up in those without a prevalent record, we identified 871 incident strokes and 1387 incident MIs, of which 8% (stroke) and 2.9% (MI) were identified in both hospital and death records. Male sex, low socio-economic status, smoking and increased body mass index were all positively associated with both stroke and MI. Compared with age and sex-matched controls, mortality for stroke and MI cases was increased both after recruitment (prevalent cases) and after hospitalization (incident cases).

## Conclusion

Information from linked coded datasets from different data providers can be combined with information collected at recruitment to identify and validate prevalent and incident acute MI and stroke.

